# Localization of RNA Pol II CTD (S5) and Transcriptome Analysis of Testis in Diploid and Tetraploid Hybrids of Red Crucian Carp (♀) × Common Carp (♂)

**DOI:** 10.3389/fgene.2021.717871

**Published:** 2021-09-09

**Authors:** Yi Zhou, La Zhu, Yu Sun, Hui Zhang, Jiaojiao Wang, Weilin Qin, Wangchao He, Luojing Zhou, Qi Li, Rurong Zhao, Kaikun Luo, Chenchen Tang, Chun Zhang, Shaojun Liu

**Affiliations:** State Key Laboratory of Developmental Biology of Freshwater Fish, College of Life Sciences, Engineering Research Center of Polyploid Fish Reproduction and Breeding of the State Education Ministry, Hunan Normal University, Changsha, China

**Keywords:** allotetraploid, RNA Pol II CTD (S5), transcriptome, chromosome, testis

## Abstract

Polyploidy occurs naturally in fish; however, the appearance of these species is an occasional and gradual process, which makes it difficult to trace the changes in phenotypes, genotypes, and regulation of gene expression. The allotetraploid hybrids (4nAT) of red crucian carp (RCC; ♀) × common carp (CC; ♂) generated from interspecies crossing are a good model to investigate the initial changes after allopolyploidization. In the present study, we focused on the changes in the active sites of the testicular transcriptome of the allotetraploid by localization of RNA Pol II CTD YSPTSPS (phospho S5) using immunofluorescence and RNA-seq data *via* bioinformatic analysis. The results showed that there was no significant difference in signal counts of the RNA Pol II CTD (S5) between the different types of fish at the same stages, including RCC, CC, 2nF_1_, and 4nAT, which means that the number of transcriptionally active sites on germ cell chromosomes was not affected by the increase in chromosome number. Similarly, RNA-seq analysis indicated that in the levels of chromosomes and 10-kb regions in the genome, there were no significant changes in the highly active sites in RCC, 2nF_1_, and 4nAT. These findings suggest that at the beginning of tetraploid origin, the active transcriptome site of 4nAT in the testis was conserved in the regions of the genome compared to that in RCC and 2nF_1_. In conclusion, 4nAT shared a similar gene expression model in the regions of the genome with RCC and 2nF_1_ with significantly different expression levels.

## Introduction

Polyploidization refers to the addition of one or more complete sets of chromosomes to the genome (Song et al., 2012; Wang et al., 2019). Polyploidy can lead to genome shock, resulting in a series of changes, such as chromosome recombination, sequence elimination, gene silencing, and changes in activation and expression levels (McClintock, 1984; Parisod et al., 2010; Liu et al., 2016; Tao et al., 2018). Changes in gene expression are directly related to the production of new phenotypes, which is of great significance in the study of polyploid evolution and breeding strategies (Pelé et al., 2018; Schiessl et al., 2019). Polyploidization and hybridization accelerate variations in phenotypes that have been reported in various organisms, such as faster growth rates, stronger antidisease abilities, and higher yields (Otto and Whitton, 2000; Zhong et al., 2019). These phenotypic changes may be induced by specific functional gene expressions in organisms. In *Populus* hybrids, changes in the expression of E3 ubiquitin ligase BIG BROTHER and growth-regulating factor were associated with organ size and leaf growth in allopolyploids (Guo et al., 2019). Although the connections between genome duplication and gene expression have been studied widely in plants, the gene expression patterns in allopolyploidy fishes are still rare (Rodriguez and Arkhipova, 2018; Wang et al., 2019). Undoubtedly, genome variations cause gene transcription to vary after polyploidization (Mason and Wendel, 2020). After genome duplication, especially in allopolyploids, different subgenomes that interact with polyploidy levels have large amounts of variation, such as mutation sites, indels, recombination, and transposition (Parisod et al., 2010; Zhang et al., 2019). In allotetraploid red crucian carp ♀ (*Carassius auratus* red var., abbreviated RCC) × common carp ♂ (*Cyprinus carpio* L., abbreviated CC), numerous chimeric genes and mutations were found, suggesting genomic incompatibilities that participate in the regulation of transcriptional changes (Liu et al., 2016). These variations in the genome contribute to transcriptome changes in allotetraploids. However, to date, there is still a lack of global information about the transcriptional activities of fish with polyploidization, particularly on the highly active sites at the genome level.

DNA-directed RNA polymerase polypeptide A (POLR2A) is the largest catalytic subunit of RNA polymerase II (RNAP II) and contains a carboxy terminal domain (CTD) composed of 52 heptapetide repeats of Tyr1–Ser2–Pro3–Thr4–Ser5–Pro6–Ser7, which is essential for polymerase activity (Corden, 2013). CTD phosphorylation at Ser5 is required for transcriptional initiation (Phatnani and Greenleaf, 2006). Thus, evaluation of RNAP II CTD repeat YSPTSPS (phospho S5; RNA Pol II CTD S5) would be helpful for understanding the global transcriptional activation in allopolyploid genomes. Allotetraploid RCC ♀ × CC ♂ can be generated from interspecies crossing. First, a diploid hybrid of RCC ♀ × CC ♂ (2n = 100, abbreviated 2nF_1_) was obtained by artificial fertilization. Subsequently, the continuous artificial fertilization of the hybrids could produce allotetraploid (4n = 200, abbreviated 4nAT) (Liu et al., 2001). Thus, this is a suitable model for unveiling the changes in transcriptional activity with polyploidization. The allotetraploid contains two subgenomes from different species that are similar to the newly formed species. In addition to allotetraploids, allodiploids can be produced as a diploid control to show the effects of polyploidization on gene expression (Liu et al., 2016).

In the present study, we used allodiploids as a diploid hybrid control to discuss the profile of global genomic transcriptional activities in allotetraploids. RNA Pol II CTD (S5) activities at the chromosome level were detected by immunofluorescence, and the staining signals were calculated for allodiploid and allotetraploid. The activated transcription sites in the genome were identified using transcriptomic data from the testes of allodiploid and allotetraploid. These results provide new insights for understanding the changes in transcriptional activity under allopolyploidization.

## Materials and Methods

### Fish Sampling

Male RCC, CC 2nF_1_, and 4nAT aged 1 year were collected from the State Key Laboratory of Developmental Biology of Freshwater Fish (Changsha, China). After euthanizing with buffered tricaine methanesulfonate (MS-222), one part of the testes from the fish was collected for testicular section preparation, and the other parts were excised for preparation of gonadal cell chromosomes. The experimenters were certified under a professional training course for laboratory animal practitioners from the Institute of Experimental Animals, Hunan, China. All experiments were approved by the Institutional Animal Care and Use Committee of Hunan Normal University (Changsha, China).

### Immunofluorescence Analysis of RNA Pol II CTD (S5) in Chromosome Spreads of Kidney Cells and Spermatocytes

The kidney and testis tissues were used to detect RNA Pol II CTD (S5) activities at the chromosome level. After adaption for 3 days, the fish were injected with concanavalin (10 μg/g body weight). Two hours post injection with colchicine (5 μg/g body weight), the kidney tissues were collected and treated with 0.075 M KCl for 60 min at room temperature. Then, the tissues were fixed with 3:1 methanol–acetic acid and dropped to precooling sections following staining with 4% Giemsa for 60 min. The separated chromosomes were blocked using 5% goat serum for 30 min at room temperature and incubated with polyclonal antibody against RNA Pol II CTD (S5; 1:100 dilution; Abcam5313, United States) after washing three times with phosphate-buffered saline (PBS). The signals were then detected using an Alexa Fluor 488-conjugated goat anti-rabbit IgG antibody (1:1,000 dilution), and 4′,6-diamidino-2-phenylindole (DAPI) was used to stain the chromosomes. The chromosomes and positive signals were analyzed using a Leica inverted DMIRE2 microscope image system (Leica, Wetzlar, Germany), and images were captured using CW4000 FISH software (Leica).

### Immunohistochemistry of RNA Pol II CTD in Testis

The testis samples were first fixed using Bouin’s fixating solution for 24 h and then dehydrated using graded ethanol solutions (75, 80, 90, and 100%). Subsequently, the tissues were treated twice with xylene and embedded in paraffin. The 6-μm-thick tissues were prepared for the sections and stained with hematoxylin and eosin.

For immunohistochemistry, the paraffin sections were dewaxed with xylene and rehydrated using graded ethanol solutions (100, 90, 80, and 75%). Antigen retrieval was performed using citrate buffer (0.01 M, pH 6.0) and treated with 10% hydrogen peroxidase in methanol. The sections were then blocked using 5% goat serum for 30 min at room temperature, followed by incubation with polyclonal antibody against RNA Pol II CTD (S5; 1:100 dilution; Abcam5131) at 4°C overnight. After washing three times with PBS, the sections were incubated with sheep anti-rabbit/rat secondary antibody–horseradish peroxidase (1:1,000 dilution) and stained with diaminobenzidine.

### Evaluation of Transcriptomic Activities Using RNA-Seq Data

Transcriptomic activities were determined using RNA-seq data. Briefly, three mature individuals from RCC, 2nF_1_, and 4nAT were used, and the testes were collected following total RNA isolation using TRIzol Reagent (Invitrogen, Carlsbad, CA, United States) according to the instructions of the manufacturer. Total RNA was analyzed on a 1.2% agarose gel, and a NanoDrop 2000 spectrophotometer (Thermo Fisher Scientific, Waltham, MA, United States) was used to evaluate the quality and concentration. In addition, the RNA integrity number (RIN) value from the Agilent 2100 Bioanalyzer (Agilent Technologies, Richardson, TX, United States) was used to determine the quality. Only samples with RIN > 8 were used for the following experiments. The sequenced cDNA libraries were constructed using TruSeq^TM^ RNA Sample Prep Kit (Illumina, San Diego, CA, United States) and sequenced on an Illumina HiSeq 6000 platform. All data were deposited in the Sequence Read Archive (SRA) database under the accession numbers SRR14463341, SRR14463346, and SRR1573262.

The raw data were processed as follows: the adaptors were trimmed, the short reads [<10 base pairs (bp)] were filtered, and low-quality reads (reads with <50% nucleotides containing bases of quality value less than 5%) were removed. The genomes of RCC and CC were used to examine transcriptomic activities at the chromosome level. The clean reads of testes from RCC were mapped to the genome of RCC,^1^ while the clean reads of testes from 2nF_1_ and 4nAT were mapped to the genomes of RCC and CC^2^ using hierarchical indexing for spliced alignment of transcripts (HISAT) (Kim et al., 2015). The regions in the chromosome were divided into 10,000 frames, and the fragments per kilobase of transcript sequence per million base pairs (FPKM) were used to evaluate the transcriptomic activities at the genome level. The results are shown as a Manhattan plot.

### Statistical Analysis

All data are presented as the mean ± standard deviation. One-way analysis of variance followed by Tukey’s test was used to compare the means between different fishes. All statistical analyses were performed using SPSS (version 17.0; SPSS Inc., Chicago, IL, United States).

## Results

### Detection of CTD Protein Signals in Chromosomes From Diploid and Tetraploid Fishes

**FIGURE 1:**
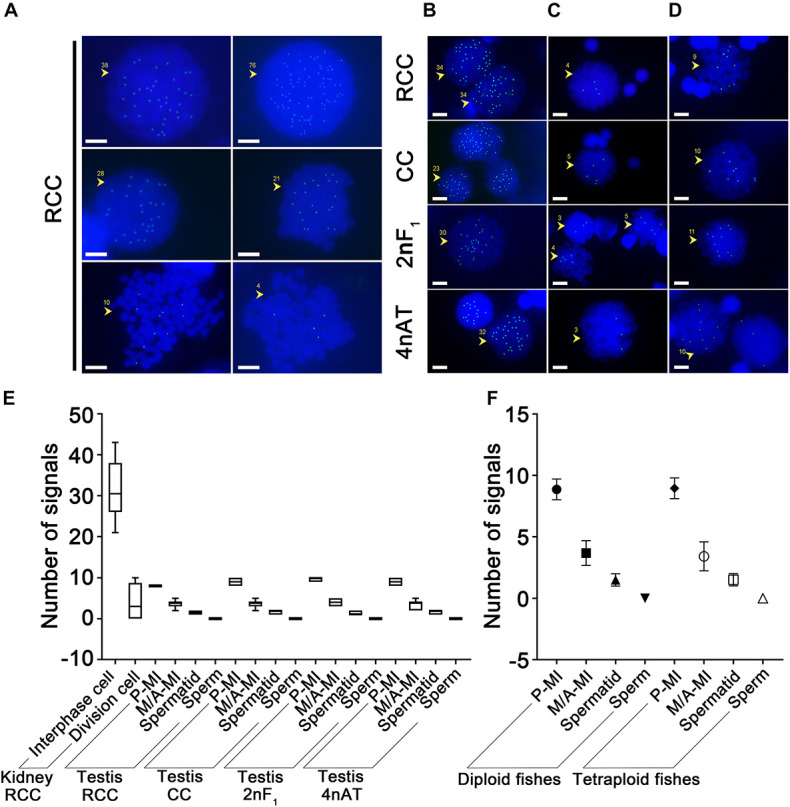
Localization of RNA Pol II CTD (S5) in the nucleus of diploid and tetraploid fishes. **(A)** The somatic cells of RCC presented 38–76 signals in interphase cells, 21–28 signals at the prophase stage of mitotic cells, and 1–10 signals in metaphase-stage cells, panels **(B–D)**, respectively. The germ cells of RCC, CC, 2nF_1_, and 4nAT presented signal distribution in the nuclei of panel **(B)** prophase of meiosis I cells (20–40 signals), **(C)** metaphase of meiosis I cells (2–5 signals), and **(D)** anaphase of meiosis I cells (8–10 signals). **(E)** Statistics of signal numbers in somatic and germ cells of all species are shown, **(F)** and these were classified as a comparison of diploid and tetraploid. The original data of the signal numbers are shown in Supplementary Tables 1, 2. P-MI, prophase of meiosis I; M-MI, metaphase of meiosis I; A-MI, antaphase of meiosis I; Spd, spermatid. The yellow arrows and numbers indicate the number of signals on the cell; bar = 5 μm.

The RNA Pol II CTD (S5) localization in the nucleus of somatic cells at different cell cycle stages of RCC was analyzed by immunofluorescence. When mitotic chromosomes agglutinated, the signals and chromosomes did not colocalize and spread throughout the nucleus, with 38–76 signals (Figure 1A). At the division stage, the number of signals decreased; approximately 21–28 signals appeared in the cells at the prophase stage (Figure 1A), and 1–10 signals were found in the cells at the metaphase stage (Figure 1A). The statistics of the signal numbers in somatic cells of RCC are shown in Figure 1E and Supplementary Table 1.

Regular signal location characteristics of RNA Pol II CTD (S5) could be detected in the spermatocytes of RCC, CC, 2nF_1_, and 4nAT following the meiotic process. Immunofluorescence analysis confirmed that RNA Pol II CTD (S5) was expressed in the nuclei of spermatocytes at different stages, with the positive signals varying in number. Small RNA Pol II CTD (S5) agglomerates and accumulations were detected during the prophase of meiosis I, and the number of signals decreased during the meiotic process and completely disappeared in sperm. There was no significant difference in the different types of fish during the division cycle of meiosis, with similar numbers of signals in the same stages. In all four species, 20–40 signals were present in the cells at the prophase of meiosis I (Figure 1B), 2–5 signals at the metaphase of meiosis I (Figure 1C), and 8–10 signals at the anaphase of meiosis I (Figure 1D). In the subsequent cell phase, spermatids showed one to two signals, and there was no signal in the sperm. The statistics of the signal numbers in germ cells of all samples are shown in Supplementary Table 2.

Statistical analysis showed that in interphase cells from kidney tissue in RCC, the highest number of RNA Pol II CTD (S5) signals was found compared to other types of cells. The number of RNA Pol II CTD (S5) signals in the dividing cells was significantly lower. Along with the development of germ cells, the signal counts decreased in the following order: prophase I, metaphase I, spermatids, and sperm. Statistics of the signal numbers in somatic and germ cells of all species are shown in Figure 1E. From these results, it is worth mentioning that there was no significant difference in the signal counts between the different types of fish at the same stages (Figure 1E). The comparison of diploid fishes and tetraploids showed that no significant difference exists between them at the same stages of spermatogenesis (Figure 1F). Binding RNA Pol II CTD (S5) is a marker for detecting transcriptionally active sites, indicating that the number of active sites on germ cell chromosomes did not change with the increase in chromosome number. This implies that the genome of 4nAT may still be transcribed according to its parental diploid pattern.

### RNA Pol II CTD Expression in Testis

**FIGURE 2:**
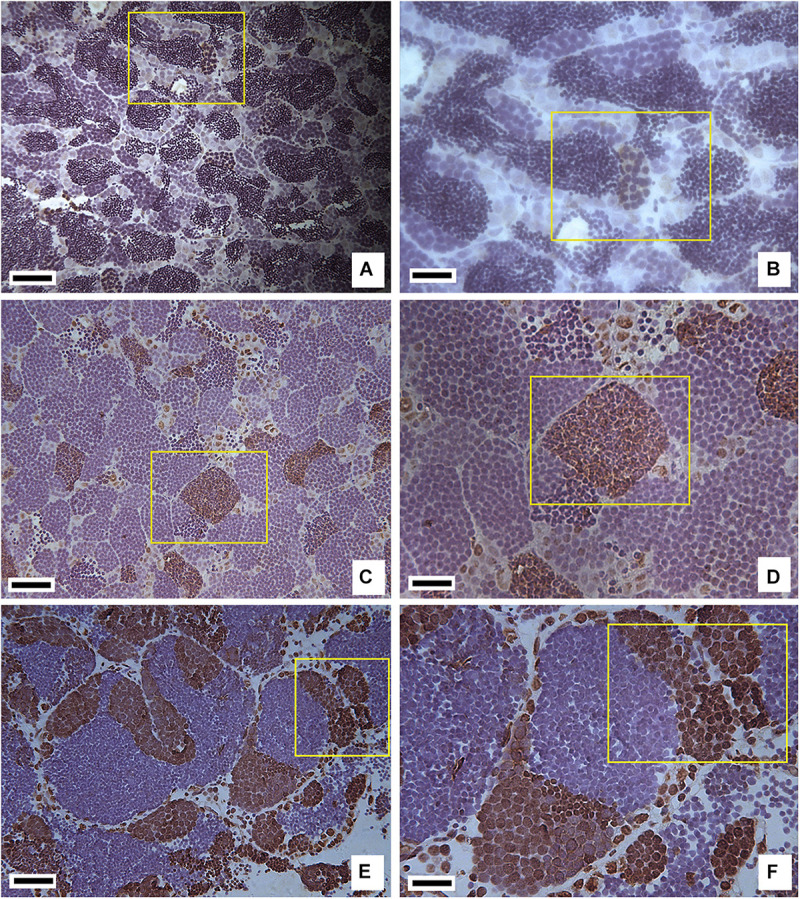
Immunohistochemistry staining of RNA Pol II CTD P(S5) in testis tissues of RCC, 2nF_1_, and 4nAT. **(A)** Immunohistochemistry staining in RCC, where the positive cells are shown in the yellow box and were inferred as spermatocytes at the early prophase stage of meiosis I. **(B)** Enlarged view of panel **(A)**. **(C)** Immunohistochemistry staining in 2nF_1_, where the positive cells are shown in the yellow box, and some of which were spermatogonia while others were a mass of primary spermatocytes in meiotic disorder. **(D)** Enlarged view of panel **(C)**. **(E)** Immunohistochemistry staining in 4nAT, where RNA Pol II CTD (S5) was highly expressed in spermatogonia and some early primary spermatocytes. **(F)** Enlarged view of panel **(E)**. In panels **(A,C,E)** bar = 25 μm; in panels **(B,D,F)** bar = 10 μm.

Immunohistochemical staining showed that RNA Pol II CTD (S5) was specifically expressed in the spermatogonia and some spermatocytes, conferring the different species with different positive antibody characteristics as they were in different developmental states. In testes from 1-year-old RCC, there were a large number of spermatids and sperm, and only several spermatocytes at the early prophase stage were positive (Figures 2A,B). The development of testes in 1-year-old hybrid 2nF_1_ was arrested during meiosis, which was accompanied by a large number of primary spermatocytes with meiotic disorders, and stained positive. Meanwhile, the testis of 2nF_1_ had some spermatogonia that stained positive, and its early primary spermatocytes and apoptotic cells were negative (Figures 2C,D). The 1-year-old hybrid 4nAT was mature, in which spermatids and sperm were observed in the testis. RNA Pol II CTD (S5) was highly expressed in spermatogonia and some early primary spermatocytes (Figures 2E,F).

### Transcriptome Analysis in Testis From RCC, Hybrid 2nF_1_, and 4nAT

**FIGURE 3:**
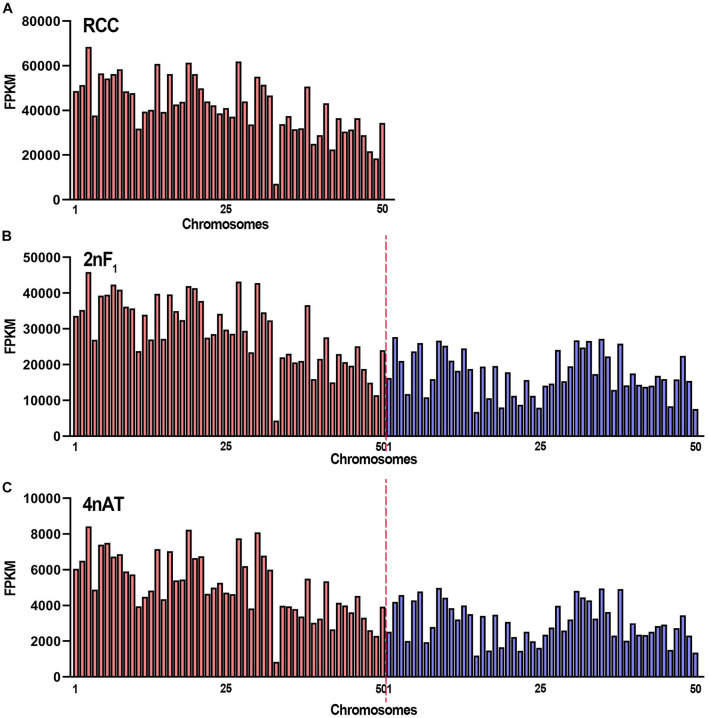
The FPKM distribution of the chromosomes in RCC, 2nF_1_, and 4nAT. **(A)** Distribution of the FPKM from RCC testicular transcriptome in RCC chromosomes. **(B)** Distribution of the FPKM from 2nF_1_ testicular transcriptome in chromosomes from RCC and common carp. **(C)** Distribution of the FPKM from 4nAT testicular transcriptome in chromosomes from RCC and CC. The red bars show the RCC chromosomes, and the blue bars show the CC chromosomes. The original data of distribution of the FPKM are shown in Supplementary Table 3.

The activity of the transcriptional sites in the genome was determined by mapping the clean reads to the genomes of RCC and CC. In RCC, the clean reads were mapped to 38,085 genes in the genome (Supplementary Table 3). All sequencing reads in 10-kb regions of the genomes were calculated using FPKM. The top three mapped chromosomes were Chr3, Chr27, and Chr19 with FPKM > 60,000, while the lowest three were Chr48, Chr49, and Chr33 with FPKM < 22,000 (Figure 3A). In hybrid 2nF_1_ and 4nAT, the fishes contained subgenomes, including the RCC and CC genomes. Clean reads were mapped to the two genomes. In hybrid 2nF_1_, the clean reads were mapped to 37,318 genes from RCC and 23,372 genes from CC (Supplementary Table 3). The top three mapped chromosomes were Chr3, Chr27, and Chr30 from RCC with an FPKM > 42,800, while the lowest three were Chr50 and Chr15 from CC as well as Chr33 from RCC with FPKM < 7,700 (Figure 3B). In 4nAT, the clean reads were mapped to 36,151 genes from RCC and 21,978 genes from CC (Supplementary Table 3). The top three mapped chromosomes were Chr3, Chr19, and Chr30 from RCC with FPKM > 8,000, while the lowest were Chr50 and Chr15 from CC and Chr33 from RCC (Figure 3C). The chromosome distributions of the highly active sites in RCC, 2nF_1_, and 4nAT were similar.

**FIGURE 4:**
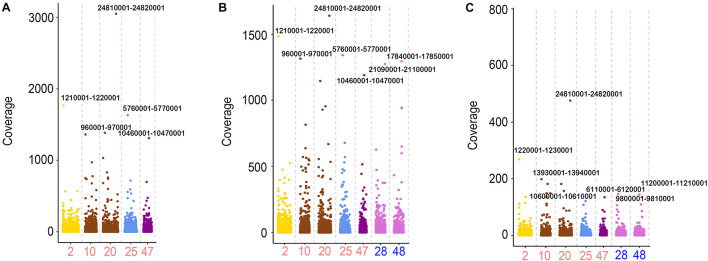
The FPKM distribution of the 10-kb regions from the chromosomes in RCC, 2nF_1_, and 4nAT. **(A)** Distribution of FPKM from RCC testicular transcriptome in the Chr2, Chr10, Chr20, Chr25, and Chr47 from RCC using the 10-kb region windows. **(B)** Distribution of FPKM from 2nF_1_ testicular transcriptome in the Chr2, Chr10, Chr20, Chr25, and Chr47 from RCC, and Chr28 and Chr48 from CC using the 10-kb region windows. **(C)** Distribution of FPKM from 4nAT testicular transcriptome in the Chr2, Chr10, Chr20, Chr25, and Chr47 from RCC, and Chr28 and Chr48 from CC using the 10-kb region windows. For each chromosome, the 10-kb regions with the highest FPKM were indicated by the locations. The original data of distribution of the FPKM are shown in Supplementary Table 3.

In addition to the analysis of chromosome levels, the distributions of the transcriptional sites were also calculated using a sliding window of 10 kb. From the statistical analysis of the sequencing reads in 10-kb regions of the genome, high similarities of the active regions were found in RCC, 2nF_1_, and 4nAT. Five chromosomes from RCC and two from CC with the highest number of active sites were calculated. In these chromosomes, the site Chr20:24810001-24820001 from RCC chromosomes had the highest expression levels in the three fishes. Chr2:1210001-1220001, Chr10:960001-970001, and Chr25:5760001-5770001 from RCC chromosomes had the highest expression levels in these chromosomes in RCC and 2nF_1_. However, the highest expression sites of CC were different from those of 2nF_1_ and 4nAT (Figure 4).

## Discussion

Although polyploid vertebrates are rare compared to plants, fish polyploidization is common in natural rivers, lakes, and seas (Leggatt and Iwama, 2003; Comber and Smith, 2004; Zhou and Gui, 2017). Several studies have shown that gene expression may not increase with polyploidization (Adams, 2007; Yoo et al., 2014). However, global information about the transcriptional activities in polyploid fishes is limited. In addition, the mechanisms of spermatogenesis in different ploidy fishes may differ, presenting the key processes to generate haploid and diploid gametes (Zhu et al., 2021). In this study, the transcriptional activities in the testes of diploid and tetraploid fishes were evaluated using RNA Pol II CTD S5 protein signals and RNA-seq data.

Previous studies have shown that RNA Pol II CTD S5 is distributed in the nuclei of mammalian cells in a dot-like pattern (Jasnovidova and Stefl, 2013). Our results showed that RNA Pol II CTD (S5) protein signals were abundant in spermatogonia and primary spermatocytes at prophase I, decreased in primary spermatocytes at metaphase I and anaphase I, and gradually disappeared in spermatids and sperm. In spermatogonia and primary spermatocytes, gene expression is activated to prepare two rounds of meiosis (Miura and Miura, 2003). Thus, it is not surprising that the RNA Pol II CTD (S5) is highly expressed during these two stages. Noticeably, the distributions of RNA Pol II CTD (S5) proteins in hybrid 2nF_1_ and 4nAT were slightly different. The RNA Pol II CTD (S5) was highly expressed in spermatogonia and some early primary spermatocytes of 4nAT, and in spermatogonia and some primary spermatocytes of 2nF_1_. This means that all experimental fish basically followed the regulation of cellular transcription activity expression according to the developmental program of germ cells; that is, the expression of RNA Pol II CTD (S5) was high in the interphase stage, low in the mitotic stage, and not expressed in sperm. Based on these results, we can also conclude that the RNA Pol II CTD (S5) protein expression in fish testes was conserved despite fish ploidy or fertility. Although it is expressed in some primary spermatocytes in 2nF_1_, it is only expressed in spermatocytes with meiotic disorders, with the early primary spermatocytes being negative. These results imply the abnormal development of germ cells in 2nF_1_ and require further investigation. Therefore, RNA Pol II CTD (S5) protein is a good marker for distinguishing the developmental status of germ cells (Rangan et al., 2009).

At the chromosome level, RNA Pol II CTD (S5) proteins showed sites with high transcriptional activities, such as dot-like signals in the nuclei of mammalian cells. The RNA Pol II CTD (S5) signals have been visualized in *Drosophila* chromosomes showing localization to sites of active transcription (Lu et al., 2019). In this study, we also determined the sites of active transcription in the fish chromosomes. Meanwhile, the interphase cells had more RNA Pol II CTD (S5) signals than metaphase cells from RCC during mitosis. During interphase, the chromatin is less condensed than it is during mitosis. The genomic DNA tends to be opened, and more sites of active transcription bind with RNA Pol II CTD (S5) for transcription (Zaborowska et al., 2016). Similarly, in meiosis of the testis, the interphase signals decreased in the order of prophase I, metaphase I, spermatids, and sperm, which follows the sequence of spermatogenesis. This is in agreement with the results of RNA Pol II CTD (S5) distribution in the testes of fish. Intriguingly, no significant differences in signal number were observed in the different ploidy fishes as well as in fertile and sterile fishes. This evidence suggests that the sites of active transcription were similar in tetraploids and diploids. Specifically, polyploidization did not change the active sites in the tetraploid.

In addition to the RNA Pol II CTD (S5) signals in the testes, the sites of active transcription were determined using RNA-seq data. At the chromosome level, the highest expression was observed in similar chromosomes and regions between 2nF_1_ and 4nAT. Chr3 and Chr30 from RCC were among the top three chromosomes in 2nF_1_ and 4nAT, suggesting that the transcriptional activities did not change significantly even when the chromosome doubled. In the 10-kb region, the highest expression regions were similar among the three fishes. For example, the site Chr20:24810001-24820001 from RCC chromosomes had the highest expression levels in the three fishes. Similar to other animals, gene expression is regulated from transcriptional initiation to RNA processing (Pandit et al., 2008; Vihervaara et al., 2018). During transcriptional initiation, the gene is controlled by *cis*- and *trans-*regulation (Morgan et al., 2020). *Cis*-regulatory elements, such as promoters, enhancers, silencers, and operators, participate in the regulation of gene transcription (Cowie et al., 2013). In hybrid fishes, two different subgenomes are present in the organism (Liu et al., 2016; Zhou et al., 2019a; Hu et al., 2021). A previous study showed the gene expression characteristic and genome instability of allotetraploids (Liu et al., 2016). The present findings reveal that the gene expression sites were similar in 4nAT and 2nF_1_. Thus, the expression changes among RCC, 2nF_1_, and 4nAT were different from the fish, while in the same kind of fish, the expression models in different regions of the genome were similar. Previously, we demonstrated that allotetraploids showed maternal-biased expression and the presence of chimera, which may be due to the effects of genome shock (Liu et al., 2016; Zhou et al., 2019b). Hybridization and polyploidization may change the genomic structures of allotetraploids, contributing to drastic changes in gene expression. Combined with the results of RNA Pol II CTD (S5) signals, these clues suggest that with the occurrence of genome duplication, the sites of single genes in the genome were duplicated, but the two sets of gene copies in genomes were not expressed simultaneously as well as in diploids. Therefore, genome duplication did not lead to the double expression of genes (Woodhouse et al., 2014; Pasquier et al., 2016). This may be explained by a special mechanism that inhibits transcriptional activity in both duplicated genomes. Epigenetic modification, chromosome condensation, and genome shock-triggered regulation of transcriptional activities may participate in this mechanism (Woodhouse et al., 2014; Alger and Edger, 2020; Zhang et al., 2021).

Taken together, the results of the present study present the comparison of transcriptional activities at the genomic level in diploid and tetraploid fishes. It was indicated that allotetraploids and diploids had no significant changes in transcriptional activities by RNA Pol II CTD (S5) signal determination and analysis of RNA-seq data from the testes. Based on the results, a comparative analysis of allotetraploid fish was performed globally, which provides new insight for future investigation at the molecular level on the mechanisms of genome duplication and polyploidization in fishes.

## Data Availability Statement

The datasets presented in this study can be found in online repositories. The names of the repository/repositories and accession number(s) can be found in the article/Supplementary Material.

## Ethics Statement

The animal study was reviewed and approved by the fish were treated humanely following the regulations of the Administration of Affairs Concerning Experimental Animals for the Science and Technology Bureau of China.

## Author Contributions

CZ and SL designed the study and carried out the analyses. YZ, LZu, YS, HZ, WH, and QL performed the technical process. CZ, YZ, LZu, and LZo prepared and drafted the manuscript. RZ, KL, and CT involved in material preparation. All authors have read and approved the final manuscript.

## Conflict of Interest

The authors declare that the research was conducted in the absence of any commercial or financial relationships that could be construed as a potential conflict of interest.

## Publisher’s Note

All claims expressed in this article are solely those of the authors and do not necessarily represent those of their affiliated organizations, or those of the publisher, the editors and the reviewers. Any product that may be evaluated in this article, or claim that may be made by its manufacturer, is not guaranteed or endorsed by the publisher.

## Footnotes

1
https://www.ncbi.nlm.nih.gov/bioproject/?term=PRJNA289059

2
https://uswest.ensembl.org/Cyprinus_carpio/Info/Index

## Supplementary Material

Supplementary Table 1 Statistics of signal numbers in somatic cells of RCC.

Supplementary Table 2 Statistics of signal numbers in germ cells of RCC, CC, 2nF_1_, and 4nAT.

Supplementary Table 3 Mapping of reads from RCC, 2nF_1_, and 4nAT to the RCC and CC genomes. The reads were available from the Sequence Read Archive (SRA) database under accession numbers SRR14463341, SRR14463346, and SRR1573262. The genomic data of RCC and CC were downloaded from the National Center for Biotechnology Information (NCBI) (https://www.ncbi.nlm.nih.gov/bioproject/?term=PRJNA289059) and Ensembl databases (https://uswest.ensembl.org/Cyprinus_carpio/Info/Index), respectively.
