# Supplementary material for: Localization of RNA Pol II CTD (S5) and Transcriptome Analysis of Testis in Diploid and Tetraploid Hybrids of Red Crucian Carp (♀) × Common Carp (♂)
Source: Front Genet. 2021 Sep 9;12:717871. doi: 10.3389/fgene.2021.717871 (PMC8458772; doi:10.3389/fgene.2021.717871)
Supplement: Supplementary Table 1 — Statistics of signal numbers in somatic cells of RCC. [file Table_1.DOCX]

Table S1 Statistics of signals number in somatic cells of RCC

| Sample | Type of cells | | | | | |
| --- | --- | --- | --- | --- | --- | --- |
|  | interphase cells | | | division cells | | |
| RCC(number of signals/number of cells counted) | 28/10 | 38/10 | 76/10 | 21-28/10 | 1-10/20 | 0/40 |
