# Supplementary material for: Localization of RNA Pol II CTD (S5) and Transcriptome Analysis of Testis in Diploid and Tetraploid Hybrids of Red Crucian Carp (♀) × Common Carp (♂)
Source: Front Genet. 2021 Sep 9;12:717871. doi: 10.3389/fgene.2021.717871 (PMC8458772; doi:10.3389/fgene.2021.717871)
Supplement: Supplementary Table 2 — Statistics of signal numbers in germ cells of RCC, CC, 2nF1, and 4nAT. [file Table_2.DOCX]

| Sample | stage | | | | |
| --- | --- | --- | --- | --- | --- |
|  | prophase I | metaphase I | antaphase I | spermatids | sperm |
| RCC(number of signals/number of cells counted) | 20-40/10 | 2-5/10 | 8-10/10 | 1-2/10 | 0/10 |
| CC(number of signals/number of cells counted) | 20-40/10 | 2-5/10 | 8-10/10 | 1-2/10 | 0/10 |
| 2nF_1_(number of signals/number of cells counted) | 20-40/10 | 2-5/10 | 8-10/10 | 1-2/10 | 0/10 |
| 4nAT(number of signals/number of cells counted) | 20-40/10 | 2-5/10 | 8-10/10 | 1-2/10 | 0/10 |

Table S2 Statistics of signals number in germ cells of RCC, CC, 2nF_1_ and 4nAT
